# Associations between sleeve gastrectomy, cholecystectomy, and gastroesophageal reflux disease in obese patients: an integrative review

**DOI:** 10.1590/0100-6991e-20253793-en

**Published:** 2025-08-18

**Authors:** GABRIEL GUERRA CORDEIRO, VINÍCIUS VASCONCELOS DO AMARAL, LUIZ ALBERTO REIS MATTOS, MATHEUS CALIXTO LEMOS, FLÁVIO KREIMER, JOSÉ GUIDO CORREIA DE ARAÚJO, ÁLVARO A. B. FERRAZ

**Affiliations:** 1 - Universidade Federal de Pernambuco, Departamento de Cirurgia - Recife - PE - Brasil; 2 - Universidade Federal de Pernambuco, Centro de Ciências Médicas - Recife - PE - Brasil

**Keywords:** Sleeve Gastrectomy, Bariatric Surgery, Cholecystectomy, Gastroesophageal Reflux Disease, Obesity, Gastrectomia Vertical, Cirurgia Bariátrica, Colecistectomia, Doença do Refluxo Gastroesofágico, Obesidade

## Abstract

**Introduction:**

Adaptations of the gastrointestinal tract after sleeve gastrectomy have been associated with an increased incidence of gastroesophageal reflux disease (GERD) and cholelithiasis. Associations between GERD and increased enterohepatic biliary circulation post-cholecystectomy have been demonstrated. The objective of this study was to analyze the possible associations between cholecystectomy, sleeve gastrectomy and gastroesophageal reflux disease in obese patients.

**Methods:**

This is an integrative review of the literature, selecting full publications, published in Portuguese, English and Spanish, between 2010 and 2023, in the databases: Web of Science, MEDLINE, LILACS, EMBASE and IBECS. The initial sample consisted of 783 studies, of which nine were selected for analysis.

**Results:**

The synthesis of the selected articles showed that in the post-sleeve gastrectomy follow-up, 32.9% of patients developed cholelithiasis, considering 17.1% in the symptomatic form, with 15.4% of the sample undergoing cholecystectomy. The development of GERD after cholecystectomy was evident in 53.6% of patients.

**Conclusion:**

Evidence from current literature suggests a relationship between GERD and patients undergoing sleeve gastrectomy and cholecystectomy procedures. The causal mechanism appears multifactorial, especially linked to anatomical, metabolic and physiological changes resulting from surgical interventions. Therefore, more studies are needed to better elucidate the outcomes and effects on the gastrointestinal dynamics that permeate this condition.

## RESUMO

**Introdução:**

As adaptações do trato gastrointestinal após a remodelação decorrente da gastrectomia vertical têm sido associadas ao aumento da incidência da doença do refluxo gastroesofágico (DRGE) e da colelitíase. Evidências correlacionam a DRGE e o aumento da circulação biliar entero-hepática pós-colecistectomia. O objetivo deste estudo consistiu em analisar as possíveis associações entre colecistectomia, gastrectomia vertical e doença do refluxo gastroesofágico em pacientes obesos.

**Métodos:**

Trata-se de uma revisão integrativa da literatura, sendo selecionadas publicações na íntegra, publicadas em português, inglês e espanhol, entre 2010 e 2023, nas bases de dados: Web of Science, MEDLINE, LILACS, EMBASE e IBECS. A amostra inicial constituiu-se de 783 estudos, dos quais nove foram selecionados para análise.

**Resultados:**

A síntese dos artigos selecionados evidenciou que no seguimento pós-gastrectomia vertical, 32,9% dos pacientes desenvolveram colelitíase, considerando-se 17,1% na forma sintomática, sendo 15,4% da amostra submetidos à colecistectomia. Evidenciou-se o desenvolvimento de DRGE após colecistectomia em 53,6% dos pacientes.

**Conclusão:**

As evidências da literatura atual sugerem uma relação entre DRGE e os pacientes submetidos aos procedimentos de gastrectomia vertical e colecistectomia. O mecanismo de causalidade parece multifatorial, sobretudo atrelado às modificações anatômicas, metabólicas e fisiológicas decorrentes das intervenções cirúrgicas. Dessa forma, mais estudos são necessários para melhor elucidar os desfechos e efeitos na dinâmica gastrointestinal que permeiam essa condição.

## INTRODUCTION

Obesity and overweight are conditions currently considered a global health problem, affecting around 59% of the world’s adult population, a high incidence. Numerous consequences for the well-being and health of the population are associated, such as risk factors for cardiovascular diseases, diabetes mellitus, neoplasms, and musculoskeletal disorders¹. In this scenario, bariatric surgery has been considered an effective and safe alternative in the treatment of severe obesity and its effects. 

Among the surgical techniques, sleeve gastrectomy is a procedure widely studied in literature, promoting restrictive and neurohormonal effects in the gastrointestinal tract. Restrictive techniques reduce gastric storage capacity, leading to early satiety and lower caloric intake². In sleeve gastrectomy, the resection of the gastric fundus contributes to the reduction in the production of ghrelin, a hormone associated with appetite induction. In addition, the reduction in gastric emptying time favors the elevation of GLP-1, an important hormone in the mechanisms of glucose metabolism and appetite reduction³. 

Despite the benefits associated with the treatment, there are inherent physiological and metabolic effects that can generate unwanted consequences. Among the main effects analyzed in the literature is the association between sleeve gastrectomy and gastroesophageal reflux disease (GERD). The mechanism of appearance or accentuation of GERD after gastrectomy seems to be multifactorial, involving physiological, anatomical, and neurological adaptations resulting from the procedure. Mandeville et al. showed a significant increase in the percentage of patients with GERD after sleeve gastrectomy, from 20% to 52% of cases^4^. In addition, factors such as obesity, dyslipidemia, and rapid weight loss resulting from bariatric surgery have been associated with gallstone formation and cholecystectomy^5^. In the post-cholecystectomy follow-up, changes in the dynamics of enterohepatic bile circulation have suggested a relationship between cholecystectomy and GERD and esophagitis^6^. Thus, the present review aims to analyze associations between GERD, cholecystectomy, and sleeve gastrectomy in the context of obesity. 

## GOAL

To analyze, through this integrative review, the possible associations between cholecystectomy, sleeve gastrectomy, and gastroesophageal reflux disease in obese patients considering the metabolic and functional dynamics of the gastrointestinal tract.

## METHODS

The present study consists of an integrative literature review, conducted in six stages: identification of the theme and formulation of the study hypothesis, establishment of selection criteria in the literature, categorization of studies, analysis of included articles, interpretation of results, and synthesis of evidence. The guiding question of the study, “What is the relationship between sleeve gastrectomy, cholecystectomy, and the worsening or appearance of GERD in obese patients?”, was based on findings in the literature suggestive of biological plausibility between these procedures and consequent adaptations of the digestive tract that lead to gastroesophageal reflux. Thus, for the selection of studies, we listed the controlled descriptors available in MesH and DeCS: Bariatric Surgery, Sleeve gastrectomy, Gastric Bypass, Obesity, Gastroesophageal reflux, Bile reflux, Cholecystectomy, and Cholelithiasis. The search strategies established consisted of their combinations in Portuguese, English, and Spanish. The databases assigned to the review were Web of Science, Medical Literature Analysis and Retrieval System online (MEDLINE), Latin American and Caribbean Literature on Health Sciences (LILACS), Excerpta Medica Database (EMBASE), and the Spanish Bibliographic Index in Health Sciences (IBECS). We chose studies published between 2010 and 2024, in view of the significant volume of publications on the subject in recent years.

The search strategy on the MEDLINE platform was: ((bariatric surgery) OR (cirurgia bariátrica) OR (cirugía bariátrica) OR (metabolic surgery) OR (bariatric surgical procedure) OR (sleeve) OR (sleeve gastrectomy) OR (vertical sleeve gastrectomy) OR (vertical gastrectomy) OR (gastrectomia vertical) OR (obesity) OR (obesidade)) AND ((cholecystectomy) OR (colelitíase) OR (cholelithiasis) OR (laparoscopic cholecystectomy)) AND ((gastroesophageal reflux) OR (bile reflux) OR (bile reflux gastritis) OR (refluxo biliar) OR (refluxo gastroesofágico) OR (gastritis) OR (reflux disease) OR (gastroesophageal reflux disease)).

We considered the level of evidence of the articles for selection, and listed case-control or cohort observational studies, as well as experimental randomized clinical trial studies. We excluded academic theses, dissertations, and duplicate articles in databases from the sample, as well as systematic or integrative literature reviews. The selection of the reviewed articles was based on the double-blind modality, minimizing subjective judgment and related biases.

**Figure 1:**
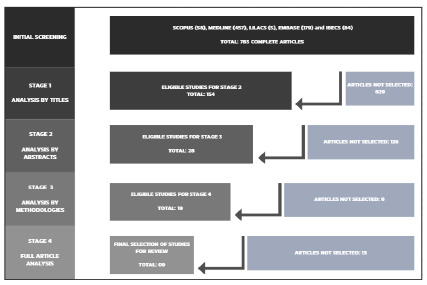
Flowchart of the steps applied to identification, analysis, and selection of the reviewed studies.

Thus, the initial sample consisted of 783 studies from the databases Scopus (58), MEDLINE (457), LILACS (5), EMBASE (179), and IBECS (84). We divided the screening and selection of articles into four stages of analysis: title, abstract, methodology, and full article. In the first stage, we excluded 629 articles due to incompatibility of the title with the theme. In the evaluation of the abstracts, we analyzed 154 studies and identified 126 that were not related to the guiding question. After analyzing the methodology in 28 studies, 19 manuscripts were eligible for the final stage. Among the 19 articles selected for full reading, nine dealt with the associations between cholecystectomy and gastroesophageal reflux disease in patients who had previously undergone sleeve gastrectomy. (Figure 1).

## RESULTS

The final sample consisted of nine studies^6^ ^-^ ^14^, among which we recorded the variables incidence of GERD after cholecystectomy, incidence of cholecystectomy after sleeve gastrectomy, and incidence of cholelithiasis after sleeve gastrectomy. Scientific evidence level III was predominant, and the selected studies were prospective and retrospective cohorts. The number of participants evaluated in the selected studies ranged from 50 to 319 patients, totaling 1,176. The more detailed specifications of each study are presented in Table 1.

Table 1a analyzes the relationship between cholecystectomy and GERD, especially by endoscopic and histopathological methods. Considering the appearance of GERD in the postoperative follow-up, among 238 patients evaluated, evidence compatible with the development of GERD was identified in 53.6% of cases after the cholecystectomy procedure. In addition, Aprea et al. showed chronic inflammation of the esophageal mucosa in 52.9% of patients and the presence of bile in 50%. In addition, Mercan et al. observed atrophy of the esophageal mucosa in 48% of patients and intestinal metaplasia in 44%. 

**Table 1a:** Cholecystectomy + GERD.

STUDY	STUDY DESIGN	SAMPLE	MÉTODO	ACHADOS
Othman et al. (2021)^6^	Retrospective cohort	62	Endoscopy; Endoscopic Biopsy; Histopathology	1. Cholecystectomy increases the incidence of biliary reflux gastropathy (61.7%) GERD (64.7%) 2. Diabetes and obesity are risk factors for duodenogastric reflux
Mercan et al. (2016)7	Retrospective cohort	50	Endoscopy; Biopsy Endoscopic; Histopathology	1. Duodenogastric reflux 48% preoperative and 78% postoperative 2. Cholecystectomy is related to atrophy of the gastric mucosa.
Aprea et al. (2012)^8^	Retrospective cohort	62	Endoscopy; Biopsy Endoscopic; Histopathology Symptomatology; Bile Reflux lndex	1. Cholecystectomy is associated with a significant risk of developing biliary gastritis - 58%
Othman et al. (2022)^9^	Case-control	64		Prevalence of biliary reflux 61.8% for patients undergoing cholecystectomy

Table 1b shows the relationship between the postoperative effects of sleeve gastrectomy and the incidence of cholelithiasis and cholecystectomy. The synthesis of the articles showed that 32.9% of patients analyzed developed cholelithiasis, of whom 17.1% had the symptomatic form. In the reviewed sample, 13.4% of the individuals underwent cholecystectomy after sleeve gastrectomy, considering the postoperative follow-up between six months and 20 years. Among the possible risk factors, Coupaye et al. showed that absolute weight loss at six months was greater in patients who developed cholelithiasis than in those who did not. The number of patients with a loss greater than 30 kg at six months post-surgery was significantly higher in those with cholelithiasis. Manatsathit et al. observed that postoperative body weight, postoperative BMI, absolute weight loss, percentage of weight loss, and rate of weight loss in both the early postoperative period and one-year post-surgery were slightly higher in patients who developed cholelithiasis and/or bile sludge compared with those who did not.

**Table 1b:** Gastrectomy + Cholecystectomy.

STUDY	STUDY DESIGN	SAMPLE	METHOD	FINDS
Coupaye et al. (2015)^10^	Retrospective cohort (Comparative study)	160	Abdominal US	Biliary findings of cholelithiasis in 28% of patients undergoing sleeve gastrectomy. Of these, 12% were symptomatic and all underwent cholecystectomy
Manatsathi et al. (2016)^11^	Retrospective cohort (Multicenter)	96	Abdominal US	Incidence of cholelithiasis 47.9%, of whom 22.9% symptomatic; 77% underwent cholecystectomy
STUDY	STUDY DESIGN	SAMPLE	METHOD	FINDS
Lucena et al. (2022)^12^	Retrospective cohort	255	Abdominal US	Biliary findings observed in 22.7% of patients, of whom 16.5% underwent cholecystectomy after sleeve gastrectomy
Vural et al. (2020)^13^	Case-control	108	Abdominal US	Cholelithiasis observed in 33% in the postoperative follow-up of sleeve gastrectomy
Dakour et al. (2016)^14^	Retrospective cohort	319	Abdominal US	Mean postoperative follow-up time of 426 days. Symptomatic cholelithiasis observed in 7.5% of the patients

USG: Ultrassonografia.

## DISCUSSION

### CHOLECYSTECTOMY AND GASTROESOPHAGEAL REFLUX DISEASEO

#### ASSOCIATIVE MECHANISM: HOW CAN CHOLECYSTECTOMY LEAD TO GERD?

The present literature shows alterations in the gastroesophageal dynamics after cholecystectomy associated with the intensification or emergence of GERD symptoms. Such symptoms seem to be related to bile reflux and, subsequently, gastropathies^15^. Following cholecystectomy, 15% to 20% of patients report new gastrointestinal symptoms or persistence of preoperative complaints^16^. The association between cholecystectomy and GERD appears to be multifactorial. The removal of the gallbladder triggers an increase in the enterohepatic circulation of bile salts, promoting duodenogastric reflux (GDR)^17^. Postcholecystectomy neurohumoral axis adaptations favor slowing of upper gastrointestinal tract motility, leading to gastric stasis, and increasing the risk of reflux^18^. Reflux of bile contents can decrease the tone of the lower esophageal sphincter, reducing its effectiveness. This mechanism may contribute to the spread of gastric acid and bile to the esophagus, exacerbating GERD, as well as cell damage, chronic inflammation, and, in severe cases, metaplasia and dysplasia.

#### CAN CHOLECYSTECTOMY BE ASSOCIATED WITH THE INCIDENCE OF GERD?

The studies presented in Table 1a analyzed the presence of GERD in endoscopic analyses and histopathological findings in the pre- and post-cholecystectomy periods. From this perspective, Othman et al. conducted an analysis of the consequences on the gastric and esophageal mucosas in a group of 62 patients who underwent cholecystectomy. Endoscopic findings of the esophageal mucosa revealed GERD in 64.7% of the cases, fluid regurgitation in 44.1%, and cardia incompetence in 35.3% of the patients. In addition, bile contents were present in the stomach of 50% of patients. Alterations in the esophagogastric mucosa have also been recorded, such as erythema, erosions, and the presence of bile⁶. Mercan et al. compared the histological findings before and after cholecystectomy in a sample of 50 patients. Preoperative endoscopies indicated GDR in 48% of patients, while postoperative endoscopies had a significantly higher incidence of GDR, present in 78% of patients^7^. 

Aprea et al. evaluated a sample of 62 patients endoscopically and histologically using the Bile Reflux Index (BRI), showing GDR and biliary gastritis in 58% of the cases six months after cholecystectomy. Clinical symptoms of GERD assessed by questionnaires were found in 41.9% of the cases^8^. In addition, Othman et al. evaluated 64 patients with refractory upper abdominal pain with symptoms of edema, belching, nausea, vomiting, and biliary regurgitation, comparing two groups: group 1, with patients without biliary tract intervention, and group 2, with patients undergoing cholecystectomy. Diabetes, obesity, elevated gastric bilirubin, and elevated stomach pH were all risk factors for bile reflux. The prevalence of biliary reflux was 16.7% and 61.8%, respectively^9^.

### SLEEVE GASTRECTOMY AND CHOLELITHIASIS

#### ASSOCIATIVE MECHANISM: HOW CAN SLEEVE GASTRECTOMY LEAD TO CHOLELITHIASIS?

The pathophysiological mechanism of the development of cholelithiasis after sleeve gastrectomy has not yet been fully elucidated. Rapid weight loss, coupled with restrictive diets induced by the procedure, increases the secretion and saturation of bile cholesterol, predisposing the formation of stones^10^. Significant gastric resection alters the composition of bile, leading to a reduction in bile acids, which are responsible for solubilizing cholesterol. The mechanism of mobilization of cholesterol from adipose tissues and excretion in bile induces reduced gallbladder motility, with subsequent stasis and concentration of prostaglandins and arachidonic acid. Such metabolic imbalance predisposes to the precipitation of cholesterol crystals, which is favorable for the nucleation and formation of cholelithiasis^21^. Another mechanism is associated with neurological impact, since lymphadenectomy and surgical maneuvers can damage the vagal innervation responsible for gallbladder motility. Vagal dysfunction is linked to reduced contractility and biliary stasis^22^.

#### CAN SLEEVE GASTRECTOMY BE ASSOCIATED WITH THE INCIDENCE OF CHOLELITHIASIS?

In the studies in Table 1b, the patients analyzed underwent abdominal ultrasonography before and after sleeve gastrectomy to screen for cholelithiasis. An important relationship was observed between rapid postoperative weight loss and a higher incidence of cholelithiasis and, consequently, cholecystectomy. Coupaye et al. analyzed 160 patients who underwent sleeve gastrectomy and found cholelithiasis in 28% of cases, of whom 12% were symptomatic and subsequently underwent cholecystectomy. Preoperative factors analyzed, as well as postoperative fat intake, did not show a significant association with the incidence of cholelithiasis after surgery. On the other hand, weight loss greater than 30 kg at six months post-surgery was significantly greater among individuals with cholelithiasis^10^. Manatsathi et al, in the primary outcome, showed the incidence of cholelithiasis in 47.9% of patients, 22.9% of them symptomatic, of whom 77% underwent cholecystectomy. In the secondary outcome, they found that weight loss in the immediate and late postoperative period was greater in patients who developed cholelithiasis^11^. Lucena et al. evaluated the effects of sleeve gastrectomy on the incidence of cholelithiasis and compared the results between the groups with cholecystectomy concomitant with the procedure and with the isolated procedure. In the sample submitted to sleeve gastrectomy alone, ultrasound findings in the gallbladder were observed in 22.7% of patients, 16.5% of whom underwent cholecystectomy^12^.

Vural et al., in a study with 108 patients, showed the development of cholelithiasis in 33% in the postoperative follow-up of sleeve gastrectomy. They found that patients who developed gallstones had greater decrease in BMI than those who did not, and this was statistically significant^13^. In a cohort study with 319 patients, Dakour et al. observed the development of symptomatic cholelithiasis in 7.5% of the patients, who subsequently underwent cholecystectomy at a mean postoperative follow-up time of 426 days^14^. 

Talha et al. studied the incidence of cholelithiasis in 1,052 patients after sleeve gastrectomy. The group with cholelithiasis had a significantly higher mean percentage of weight loss in the first postoperative year. In total, 9.7% developed cholelithiasis, 38.8% symptomatic and 61.2% asymptomatic^23^. 

#### SLEEVE GASTRECTOMY AND CHOLECYSTECTOMY, WHEN TO OPERATE CONCOMITANTLY? WHAT ARE THE BENEFITS AND RISKS?

Recent studies have suggested that bile acids may benefit energy homeostasis in obesity, with cholecystectomy being a facilitator of weight loss resulting from bariatric surgery^24^. In addition, cholecystectomy concomitant with sleeve gastrectomy has been shown to be a safe procedure that does not impair metabolic and weight results compared with sleeve gastrectomy alone. Lucena et al. evaluated the effects of sleeve gastrectomy on the incidence of cholelithiasis and compared the results between the groups of cholecystectomy concomitant with the procedure and isolated procedure. In the sample submitted to sleeve gastrectomy alone, ultrasound findings in the gallbladder were observed in 22.7% of patients, 16.5% of whom underwent cholecystectomy. They identified no statistical difference between the groups as to surgical complications, weight loss, and metabolic assessment. After cholecystectomy, the enterohepatic circulation of bile salts increases approximately twofold, making the intestine the new main reservoir. This process contributes to the increase in the synthesis of bile salts and their concentration in the bile. Although sleeve gastrectomy concomitant with cholecystectomy is associated with longer operative time, the associated procedure can reduce subsequent biliary complications in patients with cholelithiasis identified in the preoperative period, in addition to avoiding another surgical procedure later^12^.

## CONCLUSION

### ARE SLEEVE GASTRECTOMY AND CHOLECYSTECTOMY ASSOCIATED WITH THE INCIDENCE AND EFFECTS OF GERD?

The present study used the integrative review methodology, with the aim of holistically elucidating, in the light of scientific evidence, the associations between cholecystectomy, sleeve gastrectomy, and gastroesophageal reflux disease in the context of obesity. Endoscopic, clinical, and histopathological evidence from the literature suggests a close relationship between sleeve gastrectomy and gallstone formation, and, consequently, cholecystectomy, as well as the increased incidence of gastroesophageal reflux disease after this procedure. Although studies suggest this correlation, there are still gaps regarding postoperative gastrointestinal balance and its repercussions in the postoperative period. To confirm the correlation between the three factors, more targeted studies are needed. It is essential to deepen the understanding of the physiological and metabolic dynamics associated with gastroesophageal reflux in patients undergoing both cholecystectomy and sleeve gastrectomy surgeries, performed concomitantly or not.
